# Draft Genome Sequence of Enterohemorrhagic *Escherichia coli* O157:H7 Strain MC2 Isolated from Cattle in France

**DOI:** 10.1128/genomeA.01097-17

**Published:** 2017-10-05

**Authors:** Pauline Auffret, Audrey Segura, Yolande Bertin, Christophe Klopp, Olivier Bouchez, Monique Kérourédan, Delphine Bibbal, Hubert Brugère, Evelyne Forano

**Affiliations:** a Université Clermont Auvergne, INRA, MEDIS, Clermont-Ferrand, France; b Plateforme Bioinformatique Toulouse, Midi-Pyrénées UBIA, INRA, Auzeville Castanet-Tolosan, France; c GeT-PlaGe, Genotoul, INRA Auzeville, Castanet-Tolosan, France; d IRSD, Université de Toulouse, INSERM, INRA, ENVT, UPS, Toulouse, France

## Abstract

Enterohemorrhagic *Escherichia coli* (EHEC) with serotype O157:H7 is a major foodborne pathogen. Here, we report the draft genome sequence of EHEC O157:H7 strain MC2 isolated from cattle in France. The assembly contains 5,400,376 bp that encoded 5,914 predicted genes (5,805 protein-encoding genes and 109 RNA genes).

## GENOME ANNOUNCEMENT

Enterohemorrhagic *Escherichia coli* (EHEC) with serotype O157:H7 is a major foodborne pathogen responsible for serious human diseases, such as hemolytic-uremic syndrome (HUS) (1). The gastrointestinal tract of cattle is the primary reservoir of EHEC O157:H7, which can be transmitted from cattle to humans by means of contaminated meat or unpasteurized milk (1). In this report, we present the draft genome sequence of EHEC O157:H7 strain MC2 isolated in France from cattle on a farm in which persistent shedding has been observed (2). Using real-time PCR (3, 4), MC2 was confirmed to belong to serotype O157:H7 and to carry key virulence genes of EHEC O157:H7:*eae*-γ1 encoding intimin (bacterial attachment to human epithelial cells) and *stx1* and *stx2* encoding Shiga toxins (responsible for HUS).

We sequenced *E. coli* MC2 because a better understanding of the nature of *E. coli* O157:H7 colonization of cattle is required to develop more effective preharvest food safety practices and to reduce the contamination of food of bovine origin.

*E. coli* MC2 was cultured on Luria-Bertani medium, and the genomic DNA was extracted using the DNeasy blood and tissue kit (Qiagen). Whole-genome sequencing was performed at the GeT-PlaGe core facility (INRA Toulouse, France [http://get.genotoul.fr/]). DNA-seq libraries were prepared according to Illumina’s protocols (Illumina TruSeq Nano DNA LT library prep kit). DNA-seq experiments were performed on an Illumina MiSeq instrument using a paired-end read length of 2 × 250 bp with the Illumina MiSeq reagent kit v2. The raw reads were stored in NG6 (5) and quality checked using FastQC (6). They were assembled with SPAdes v3.1.1 (7) using standard parameters and annotated with the NCBI Prokaryotic Genome Annotation Pipeline (8).

The resulting assembly consisted of 265 contigs. The MC2 genome comprised 5,400,376 bp with a G+C content of 50.34% and contained 5,805 protein-encoding genes (4,560 proteins with predicted functions and 4,144 assigned to cluster of orthologous groups (COG) of proteins using BLASTp) and 109 RNA genes. The contigs were aligned to the *E. coli* Sakai genome (NCBI Reference Sequence NC_002695) and pO157 plasmid (NC_002128) using Mauve (v2.3.1). The EHEC strain Sakai, isolated from a large-scale outbreak in Japan, is a standard reference for comparative genomic studies of O157:H7 *E. coli* (9). This analysis revealed that 94.19% of pO157 was covered by MC2 contigs (99.93% of homology). A total of 71 out of the 85 genes carried by plasmid pO157 were present in the MC2 assembly (average homology, 99.74%), suggesting that MC2 carries at least one major plasmid homolog to pO157. Comparison of MC2 and Sakai genes using BLASTn (70% coverage and identity cutoff) demonstrated that 4,608 of the MC2 functional genes were present in *E. coli* O157:H7 Sakai, showing an average homology of 99.91%. Only 803 genes were unique to MC2. The high homology between the two genomes suggests that the bovine *E. coli* strain MC2 possesses the characteristic of an EHEC strain potentially responsible for clinical cases.

### Accession number(s).

This whole-genome shotgun project has been deposited at DDBJ/ENA/GenBank under the accession number NJDB00000000. The version described in this paper is version NJDB01000000.
